# Optimization of Peripheral Vascular Sizing with Conductance Guidewire: Theory and Experiment

**DOI:** 10.1371/journal.pone.0168886

**Published:** 2017-01-03

**Authors:** Hyo Won Choi, Zachary C. Berwick, Matthew S. Sulkin, Christopher D. Owens, Ghassan S. Kassab

**Affiliations:** 1 The California Medical Innovations Institute, Inc., San Diego, California, United States of America; 2 3DT Holdings LLC, San Diego, California, United States of America; 3 Department of Vascular Surgery, UCSF, California, United States of America; "INSERM", FRANCE

## Abstract

Although the clinical range of interventions for coronary arteries is about 2 to 5 mm, the range of diameters of peripheral vasculature is significantly larger (about 10 mm for human iliac artery). When the vessel diameter is increased, the spacing between excitation electrodes on a conductance sizing device must also increase to accommodate the greater range of vessel diameters. The increase in the excitation electrodes distance, however, causes higher parallel conductance or current losses outside of artery lumen. We have previously shown that the conductance catheter/guidewire excitation electrode distances affects the measurement accuracy for the peripheral artery lumen sizing. Here, we propose a simple solution that varies the detection electrode distances to compensate for parallel conductance losses. Computational models were constructed to simulate the conductance guidewire with various electrodes spacing combinations over a range of peripheral artery lumen diameters and surrounding tissue electrical conductivities. The results demonstrate that the measurement accuracy may be significantly improved by increased detection spacing. Specifically, an optimally configured detection/excitation spacing (i.e., 5-5-5 or an equidistant electrode interval with a detection-to-excitation spacing ratio of 0.3) was shown to accurately predict the lumen diameter (i.e., -10% < error < 10%) over a broad range of peripheral artery dimensions (4 mm < diameter < 10 mm). The computational results were substantiated with both *ex-vivo* and *in-vivo* measurements of peripheral arteries. The present results support the accuracy of the conductance technique for measurement of peripheral reference vessel diameter.

## Introduction

Peripheral artery disease (PAD) is a common defect in the circulatory system in which occluded peripheral arteries reduce blood supply to the lower extremities such that the limbs are not adequately perfused. The most common cause of PAD is atherosclerosis [1] which is a disease that reduces the lumen and compromises blood flow. Clinical treatment of PAD is essential to reduce cardiovascular morbidity and mortality and provides better quality of life. For proper PAD diagnosis, it is essential to precisely assess the severity of peripheral disease of vessel lumen.

The conductance technique has been used for determination of lumen cross-sectional area (CSA) of coronary and peripheral arteries [2–5]. To accurately determine the CSA of the vessel lumen and the parallel conductance (G_p_) through the vessel wall and surrounding tissue, a two bolus injections of saline solutions with different salinities (normal and half normal) has been proposed [3,4]. In this approach, an underlying assumption is that the parallel conductance is constant over the injections of two different saline solutions. This assumption may be challenged when G_p_ becomes excessively high (e.g., when over 90% of current is lost through the vessel wall and surrounding tissue). As we have recently shown, G_p_ is strongly influenced by the distance (L_EE_) between the excitation electrodes [6] so that the accuracy of lumen sizing can be deteriorated by a greater G_p_ due to a larger excitation spacing. As the range of vessel diameter (D) of interest becomes larger, the distance between the excitation electrodes must increase to maintain the cylindricity of electric field such that L_EE_ > 2D [3]. For example, the reference vessel diameter (RVD) for peripheral interventions may be 10 mm (e.g., human iliac artery) and hence L_EE_ should be approximately 20 mm to maintain a cylindrical electric field to obey Ohm’s law for accurate calculation of lumen CSA. Hence, the design of the conductance guidewire requires a compromise between L_EE_ to be large enough to maintain a cylindrical electric field and G_p_ to be small enough to maintain the accuracy of the two injection method. To our knowledge, this optimization has not been performed.

In fact, our previous study [6] has shown that the unfavorable impact of parallel conductance on the accuracy of conductance guidewire measurement can be decreased by the combination of 1) a smaller excitation electrodes spacing (L_EE_) for a relatively smaller range of coronary RVDs and 2) superficial anatomical positions with surrounding fatty tissue of coronary arteries. Unfortunately these conditions (smaller range of dimensions and superficial epicardial coronary arteries) may not hold for the peripheral arteries. To address this issue, the design of distance between detection electrodes (L_DD_) may be a practical approach to mitigate the effect of parallel conductance over luminal conductance for peripheral vessel (i.e., larger L_EE_ due to larger RVD and anatomical positions leading to more current leakage).

Here, we hypothesize that an increase in detection electrodes spacing (larger than that used for coronary measurements) will amplify the signal voltage and hence the lumen conductance relative to surrounding tissue increase to compensate for the increased parallel conductance. To confirm the positive effect of increase in detection spacing on measurement accuracy, we performed computer simulations over various guidewire-vessel-tissue configurations and examined the role of detection to excitation spacing in determining the peripheral artery lumen area. We optimized the configuration of excitation/detection electrodes that provides the highest accuracy of lumen sizing over a wide range of peripheral artery diameters.

## Methods

### Computational Domain

**Fig 1:**
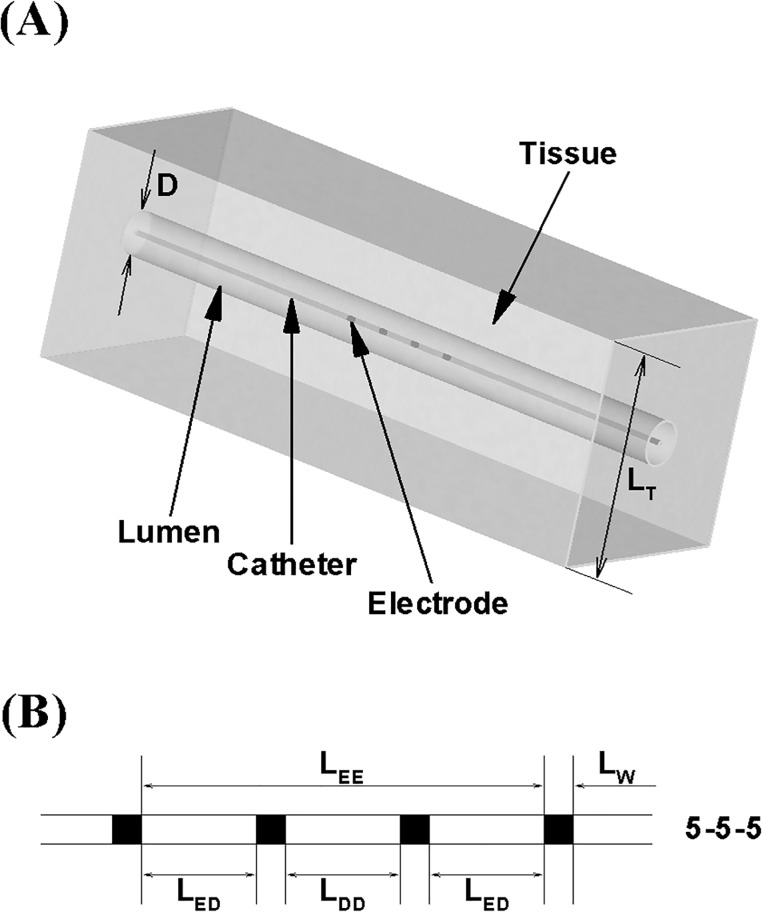
**(A)** Schematic of computational domain that simulates a peripheral vessel surrounded by conductive medium to mimics skeletal muscle tissue. L_T_ and D denote the total tissue thickness and the lumen diameter, respectively. **(B)** Configurations of detection/excitation electrodes implemented on the conductance catheter. L_EE_, L_DD_, L_ED_, and L_W_ designate the inter-excitation, inter-detection, excitation-detection electrodes distance, and electrode width, respectively. Four different L_DD_ were constructed (i.e., 7-1-7, 6-3-6, 5-5-5, and 4-7-4).

Computer simulations were performed in a 3-D geometry that models a peripheral vessel surrounded by the conductive medium to mimic the surrounding tissue as shown in Fig 1A. The total thickness of surrounding tissue L_T_ was assumed to be 3.5 cm based on our previous study [6]. The depth of a blood vessel L_D_ was modeled as 12.7–15.7 mm which is commensurate with the deepest limit of blood vessel depths modeled previously [6] that can have the most unfavorable impact on the measurement accuracy. The entire domain including conductance guidewire, vessel, and tissue was assumed to be cylindrically axisymmetric similar to previous conductance catheter studies [3,7,8].

### Guidewire Configuration

The conductance guidewire was modeled such that it has 0.9 mm diameter (corresponding to 0.035” device) and four electrodes with 1 mm width, L_W_. The conductance guidewire utilized for ex-vivo and in-vivo testing consists of a 260 cm body length, hydrophilic coated workhorse wire (mechanically similar to Wholey) comprised of a stainless steel core, atraumatic tip and containing 4 electrodes and a thermistor to automate measurements through a touch screen console. A wide variety of inter-electrodes distances were modeled to determine the effect of detection/excitation spacing on the measurement accuracy. Specifically, four different excitation (i.e., L_EE_ = 14–17 mm) and detection spacing (i.e., L_DD_ = 1–7 mm) were modeled, respectively; as illustrated in Fig 1B.

### Governing Equation and Numerical Modeling

The electric field in the vessel lumen and surrounding tissue generated by the conductance guidewire was obtained by solving the Poisson’s equation:

∇∙(σ∇∅)=I, (1)

where σ, ϕ, and I denote electric conductivity, electric potential, and driving current, respectively. For the vessel lumen, 1.6 and 0.8 S/m of electrical conductivity was assumed for normal and half-normal saline injections, respectively. For the surrounding medium, the electrical conductivity was calculated such that it is commensurate with 0.1–0.2% saline solution according to the linear relationship between salinity and electrical conductivity (i.e., 0.178–0.356 S/m).

As described in detail in the previous studies [3,4], CSA and parallel conductance in the two injection approach were analytically determined using the following equations:

$$CSA=L_{DD}∙\frac{G_{2}-G_{1}}{\sigma_{2}-\sigma_{1}},$$ (2a)

$$G_{p}=\frac{\sigma_{2}∙G_{1}-\sigma_{1}∙G_{2}}{\sigma_{2}-\sigma_{1}},$$ (2b)

where G, G_p_, and L_DD_ denote total conductance, parallel conductance, and detection electrodes spacing, respectively, and subscripts 1 and 2 designate the two different salinities (normal and half normal saline), respectively. ANSYS FLUENT (version 14.5, ANSYS, Inc.) was used to solve the governing equations.

### Ex-vivo Measurements

Arteries from Yorkshire swine (90–110 kg) were provided by Sierra Medical, Whittier, CA. They obtain specimens from a USDA approved slaughterhouse. The procedure for *ex-vivo* measurements was consistent with our previous study [6]. Briefly, isolated segments of arteries were obtained from a slaughter house and rinsed with saline. The segments were then placed in a bath of either 0.1% or 0.2% NaCl and perfused with two different saline solutions (i.e., 0.9% and 0.45%) for two conductance measurements. For each vessel, the conductance and diameter prediction error was obtained using 2 guidewires of a given spacing (i.e., 6-3-6 and 5-5-5). We ruled out the smallest and largest detection spacings (i.e., 7-1-7 and 4-7-4) that may lead to under- (i.e., < -10%) or over-estimation (i.e., > 10%) as suggested by the computer simulations. Measurements were taken in quadruplicate and compared to ultrasound (control, taken in triplicate). This was performed in the 0.1% and 0.2% external bath saline concentrations for each vessel. A total of 7 vessels (D = 4.3–11.1 mm) were tested with each guidewire for 12 data points per configuration at each external saline concentration.

### In-vivo Measurements

All animal experiments were performed in accordance with national and local ethical guidelines, including the Principles of Laboratory Animal Care, the Guide for the Care and Use of Laboratory Animals and the National Association for Biomedical Research, and an IACUC at California Medical Innovations Institute approved the protocol regarding the use of animals in research. *In-vivo* measurements were performed in four normal domestic swine (Yorkshire swine, 60–80 kg, male) from Oak Hill Genetics, Ewing Township, IL. Animals were hosed in climate controlled rooms under veterinary supervision and allowed to acclimate for at least 3 days prior to acute procedures. They fed standard maintenance diet from Envigo, Indianapolis, IN. Animal preparation procedure was consistent with our previous study [9]. Briefly, animals were sedated using telazol (2 mg/kg), ketamine (1 mg/kg), and xylazine (1 mg/kg) with anesthetic maintenance through intubation and ventilation with 100% oxygen and 1–2% isoflurane. At the end of the procedure, animals were euthanized by intravenous injection of saturated KCl under deep anesthesia. This is consistent with recommendations of the American Veterinary Medical Association Panel on Euthanasia.

A comparison of blood vessel sizing *in vivo* is confounded by several variables including matching of vessel location, state of vessel (e.g., spasms), and injection pressure. To address these issues, phantoms of known dimension (vascular access sheaths) were used to determine absolute accuracy of ultrasound (US), quantitative angiography (QA), and conductance guidewire. The sheaths are electrically insulated and rigid and hence represent the gold standard for conductance measurements. Vascular sheaths of multiple diameters (12F, 14F, 16F and 18F) were inserted into the peripheral vessels and advanced from the femoral artery. The conductance measurements (n = 7 wires, 4 animals) were taken with the guidewire placed ~5 cm inside the body. The saline bags were pressurized externally in order to achieve steady state infusions and blood volume displacement of the larger sheaths with 0.45% and 0.90% saline. Effects of temperature were negligible given the length and volume of injection; i.e., infusion remained nearly at room temperature. US measurements (n = 4 animals) were obtained simultaneously with conductance measurements.

Following US and conductance measurements, QA (n = 3 animals) was performed in triplicate with the wire inside of the sheath and calibrated using an external radiopaque ruler. The location of measurement for US and QA were the center point between detection electrodes and taken in triplicate during peak injection of either saline (US) or contrast (QA). Data obtained in triplicate for each modality were averaged first before expression as grouped average and compared against known phantom diameters. The inner diameters of phantoms were confirmed using a microscope and a digital micrometer. For comparison of the measurement accuracy, the Bland-Altman analyses were carried out for the three different CSA measurement methods (i.e., conductance, US, and QA) relative to a gold standard (microscope measurements of phantom sheaths).

In addition to the phantom measurements made in vivo, we also sized peripheral arteries using conductance guidewire, US and QA. A sheath was placed in the right femoral artery and the sizing guidewire was advanced to the iliac, left common femoral artery or superficial femoral artery (SFA) of the counter lateral side. An arteriogram was taken using contrast injection at the location of interest as identified by an x-ray ruler along with anatomical landmarks. Similar to in vivo measurements made with phantom described above, repeat guidewire sizing measurements were made using injections of 0.45% and 0.9% NaCl solutions through the guide catheter simultaneously with B-mode US measurements. The detection electrodes were visible with US and the midpoint of which marked the point of measurements. After sizing, the animal was euthanized by an intravenous bolus of potassium chloride and heart removal.

In the present study, three different measurement methods (i.e., conductance, US, and QA) were used to determine the lumen diameter of blood vessels. To assess the accuracy (for sheaths) or agreement (for vessels) of various methods, we used the Bland-Altman analysis to compare the conductance method to two routine sizing imaging modalities (US and QA) used clinically. In a Bland-Altman scatter diagram, we plotted the difference in diameter from two measurements as a function of their means to indicate the precision and bias of the method.

## Results

### Simulations

**Fig 2:**
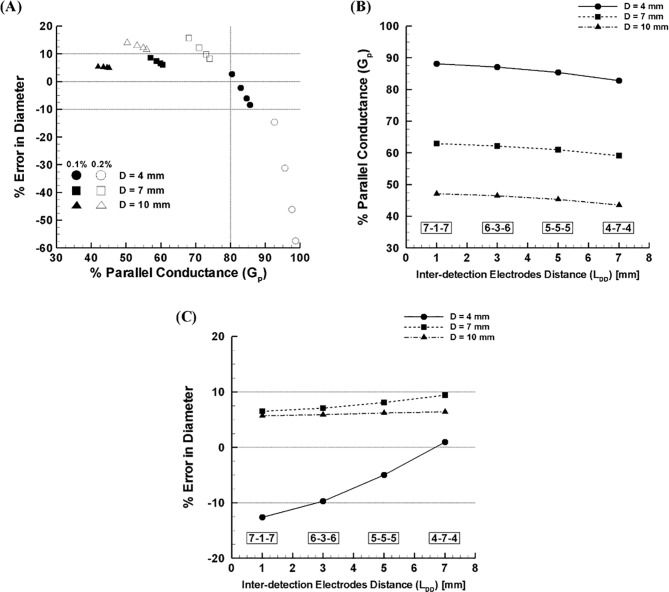
**(A)** Error in diameter as a function of parallel conductance for three different peripheral artery diameters (i.e., D = 4, 7, and 10 mm) with a range of surrounding tissue conductivities (i.e., σ_T_ = 0.178–0.356 S/m or 0.1–0.2% saline solution) using four different catheter configurations (i.e., 7-1-7, 6-3-6, 5-5-5, and 4-7-4 or L_DD_ = 1–7 mm). A group of four symbols represent 7-1-7, 6-3-6, 5-5-5, and 4-7-4; from right to left (e.g., first symbol in each group denotes the 4-7-4 configuration). **(B)** Parallel conductance with inter-detection electrodes distance for various peripheral artery diameters (i.e., D = 4, 7, and 10 mm) with surrounding tissue conductivity of 0.2 S/m. **(C)** Error in diameter with inter-detection electrodes distance for various peripheral artery diameters (i.e., D = 4, 7, and 10 mm) with surrounding tissue conductivity of 0.2 S/m.

The percent error in diameter prediction was calculated as a function of the percent parallel conductance for three different peripheral artery diameters (i.e., D = 4, 7, and 10 mm) with a range of surrounding tissue conductivities (i.e., σ_T_ = 0.178–0.356 S/m or 0.1–0.2% saline solution) using four different guidewire configurations with an identical excitation spacing or L_EE_ = 17 mm (i.e., 7-1-7, 6-3-6, 5-5-5, and 4-7-4 or L_DD_ = 1–7 mm). Regardless of the guidewire configurations considered, the results demonstrated that the absolute value of error increases as the conductivity of surrounding tissue increases (compare two different colors of an identical symbol, Fig 2A) and such pattern is more discernable with a smaller diameter (i.e., D = 4 mm). Also, the magnitude of the parallel conductance or the percent parallel conductance was shown to decrease as the vessel diameter increased regardless of the guidewire configurations and tissue conductivities (compare three different symbols with an identical color, Fig 2A).

One finding is that the magnitude of error does not have a simple linear correlation with the percent parallel conductance (compare black circular symbols with rectangular and triangular symbols, Fig 2A). In other words, the diameter was shown to be over-estimated for the percent parallel conductance < 80% while it was under-estimated as the percent parallel conductance exceeds 80%. Thus, the error magnitude was shown to significantly increase with decrease in detection spacing for the excessively high parallel conductance (i.e., > 90%) while it was shown to not change substantially with decrease in detection spacing for the moderate range of parallel conductance (i.e., < 75%).

Since the primary measurement error stems from the parallel conductance, the intent is to minimize the effect of parallel conductance on the accuracy. A larger detection spacing can reduce the parallel conductance by increasing the potential gradient or enhancing the detection signal strength measured by the pair of detection electrodes (Fig 2B and 2C). To determine how the detection spacing affects the measurement accuracy, the percent error in diameter was calculated for four different electrode configurations which had an identical excitation length or L_EE_ = 17 mm but varied detection spacing or L_DD_ = 1–7 mm over the various surrounding tissue conditions (i.e., σ_T_ = 0.178–0.356 S/m or 0.1–0.2% saline solution) and lumen diameters (D = 4–10 mm).

**Fig 3:**
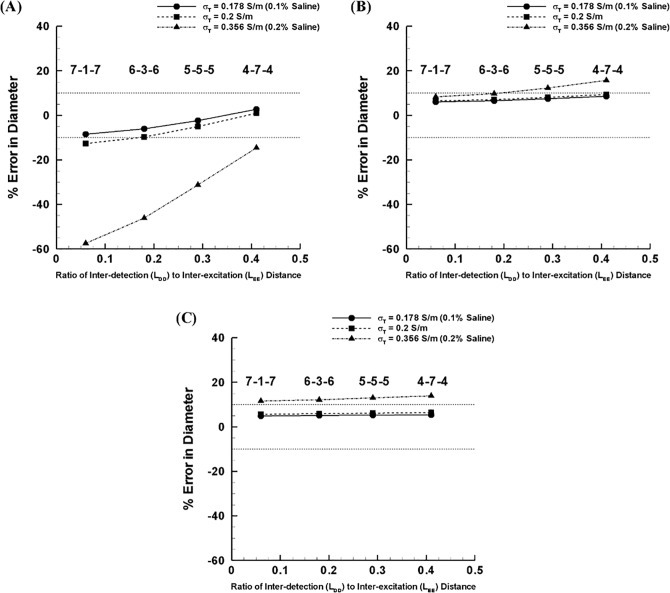
Error in diameter with the ratio of detection to excitation spacing subject to various electrical conductivities of surrounding tissue (i.e., electrical conductivity corresponding to 0.1%– 0.2% saline solutions) for **(A)** D = 4 mm, **(B)** D = 7 mm, and **(C)** D = 10 mm.

For D = 4 mm, the results show that increased detection spacing significantly decreases the error for all simulation cases of parallel conductance considered (Fig 3A). The effect of detection-to-excitation length ratio on sizing error, however, was much less significant in larger vessels. For D = 7 mm, the increase in detection spacing can result in increase in percent error to an unacceptable level (> 10%) at ratio greater than 0.3 (Fig 3B). For the largest vessel diameter considered (D = 10 mm), the impact of detection spacing was shown to be insignificant regardless of surrounding tissue conditions (Fig 3C).

**Fig 4:**
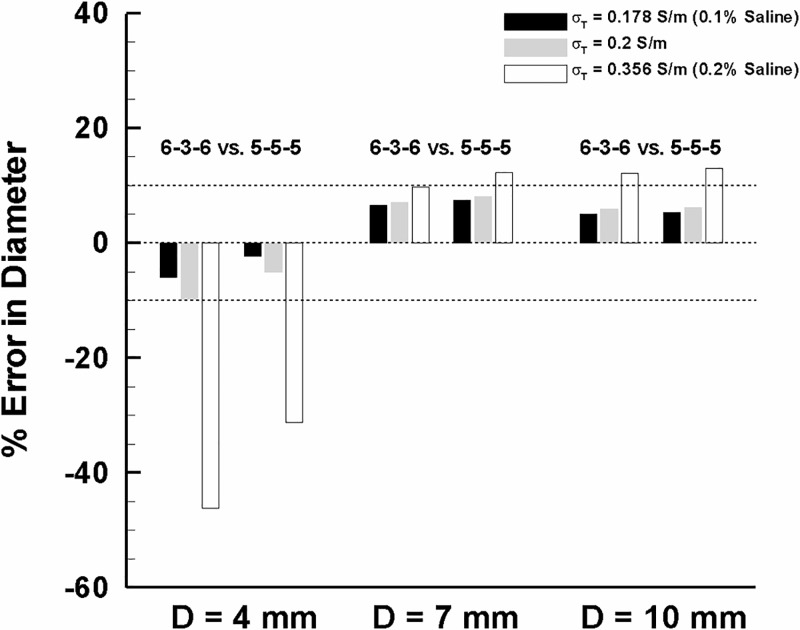
Measurement accuracy improvement over a wide range of peripheral artery diameters (i.e., D = 4–10 mm) and surrounding tissue conditions (i.e., σ_T_ = 0.178–0.356 S/m or 0.1%– 0.2% saline solutions).

Since there is an optimal detection-to-excitation length ratio (i.e., 0.2–0.3) that can confine the error magnitude, we ruled out the smallest and largest detection spacing (i.e., 7-1-7 and 4-7-4) which might lead to an undesirable under- (i.e., < -10%) or over-estimation (i.e., > 10%). We focused on the two most promising detection spacings (i.e., 6-3-6 and 5-5-5) for further assessment over the range in vessel sizes and surrounding tissue conditions considered. The results indicate that the 5-5-5 spacing design is the best compromise to minimize sizing error for the full range of vessel dimensions considered (Fig 4). For the smallest vessel size (i.e., D = 4 mm), the error was reduced from -46.1% to -31.2% as indicated in Fig 4 for the extreme (higher than physiological) conductive surrounding medium (i.e., σ_T_ = 0.356 S/m or 0.2% solution). The accuracy was also significantly improved (i.e., -9.7% to -5%) for the moderate medium conductivity (i.e., σ_T_ = 0.2 S/m). For the larger vessel sizes (i.e., D = 7–10 mm), however, the error was not significantly changed for the two different spacing configurations.

### Ex-vivo Vessels

**Fig 5:**
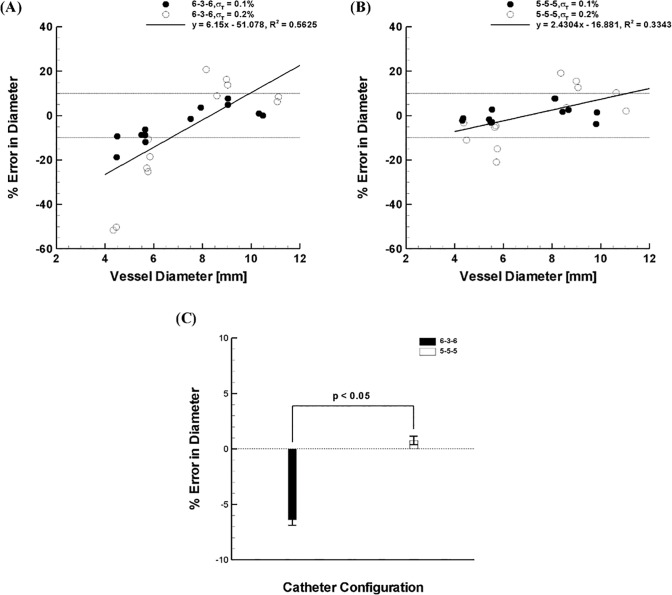
*Ex-vivo* measurements of various peripheral artery lumen diameters (> 4 mm) subject to two different saline bath concentrations (i.e., 0.1% and 0.2% saline) with two different catheter configurations (i.e., **(A)** 6-3-6 vs. **(B)** 5-5-5). **(C)** Overall error for two different catheter configurations. Error bars indicate standard error of the mean (SEM). A total of 7 vessels (N = 7) were tested with each guidewire for 12 data points (n = 12) per configuration at each external saline concentration.

To validate the trends from the computer simulations, the diameter measurements were performed *ex vivo* using peripheral artery segments with various lumen diameters for two different catheter configurations (i.e., 6-3-6 vs. 5-5-5, Fig 5). A least products regression analysis showed that the lumen size prediction tends to vary from under- to over-estimation with increasing vessel diameters by both catheter configurations although such tendency is more substantial (i.e., higher proportional bias) for the 6-3-6 configuration. Similar to the computer simulations, the 5-5-5 configuration demonstrated significant improvement in sizing accuracy with minimal changes in measurement error (i.e., p < 0.05, Fig 5C). These improvements in accuracy with 5-5-5 spacing was particularly evident in smaller vessels (i.e., D = 4–6 mm, Fig 5A and 5B). This is also consistent with observations from the computer simulations.

### In-vivo Studies

#### Sheaths

**Fig 6:**
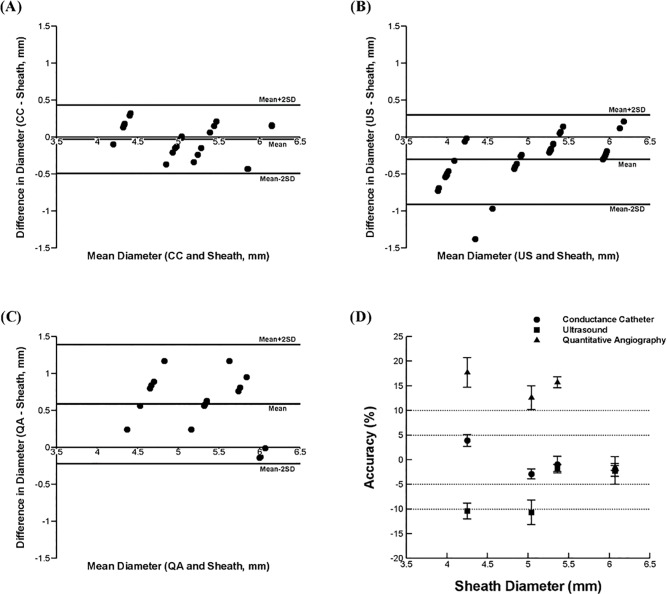
Bland-Altman analysis for pairwise comparisons of mean diameter for the conductance & sheath **(A)**, US & sheath **(B)**, QA & sheath **(C)**, and accuracy of measurements with conductance catheters over ultrasound and quantitative angiography **(D)**. Error bars indicate standard error of the mean (SEM). The conductance and US measurements were made in 4 animals (N = 4) for total of 24 (n = 24) and 39 (n = 39) data points, respectively. QA measurements were made in 3 animals (N = 3) for total of 18 (n = 18) data points.

The Bland-Altman analyses expressing the differences between the diameter measurements of each modality and the sheath *in vivo* against their means are shown in Fig 6. In a scatter diagram, the mean difference of the diameter was shown to be -0.03±0.23mm for conductance, -0.3±0.3mm for US, and 0.59±0.4mm for QA, respectively as compared to the sheath. Data obtained from conductance, US, and QA measurements using the sheath were also expressed as the difference between the modality and the sheath to determine the percent accuracy of each modality (Fig 6D). The average accuracy in lumen or inner dimeter for 12F, 14F, 16F, and 18F sheath (correspond to 4 to 6 mm of outer diameter) was shown to be 3.9±1.2%, -2.9±1.0%, -1.0±1.7%, and -2.2±2.8% for the conductance method, -10.4±1.6%, -10.7±2.5%, -1.7±0.7%, and -2.3±1.1% for the US, and 17.7±3.0%, 12.6±2.4%, -15.7±1.1%, and -1.5±0.7% for the QA, respectively.

#### Vessels

**Fig 7:**
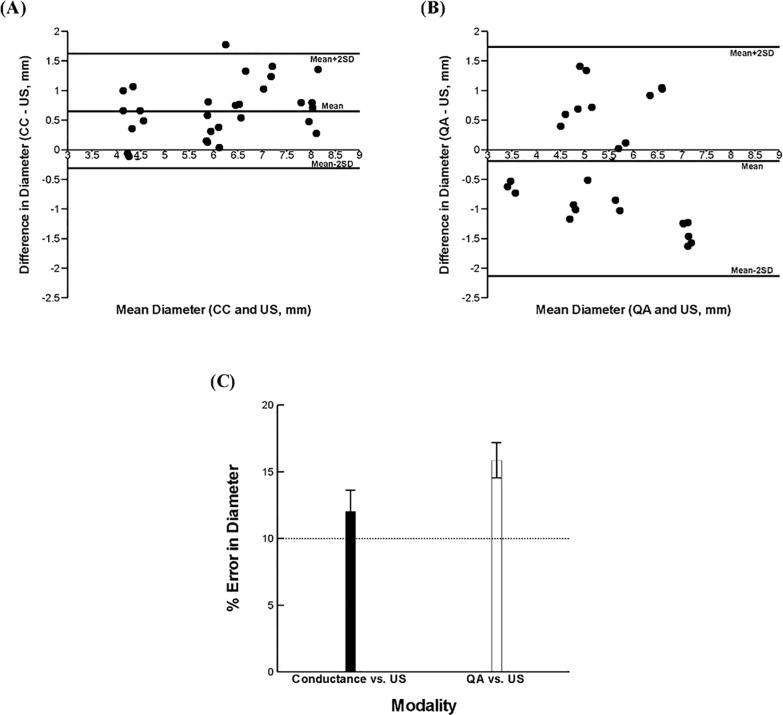
Bland-Altman analysis for pairwise comparisons of mean diameter for the conductance & US **(A)** and QA & US **(B)**. **(C)** Measurement error of conductance catheters and quantitative angiography over ultrasound. Error bars indicate standard error of the mean (SEM). The measurements were taken for 30 data points (n = 30) with each modality.

Bland-Altman analyses representing the differences in diameter measurements of peripheral vessels between two different modalities conducted simultaneously *in vivo* against their means are shown in Fig 7A and 7B. The mean difference of the diameter was shown to be 0.65±0.48mm between conductance and US and -0.19±0.97mm between QA and US. The measurement differences were 12.1±1.56% between conductance method and US and 15.9±1.3% between QA and US (Fig 7C), respectively.

## Discussion

The results demonstrate that an optimal ratio of detection to excitation distance can minimize the diameter measurement errors in a wide range of vessel diameters by enhancing the lumen conductance relative to parallel conductance. In order for the two injection method to provide accurate sizing, the parallel conductance must be minimized (i.e., < 90%, Fig 2A). Increase of detection spacing can improve the measurement accuracy by enhancing the signal-to-noise ratio and thus minimizing the unfavorable influence of excessive parallel conductance on the accuracy at a given excitation electrode spacing especially when the vessel diameter of interest is smaller (Fig 2). Unlike the coronary arteries, a larger detection spacing is clinically acceptable since the segment length of reference vessel diameter is significantly larger and hence an average over the 5 mm length is desirable. In the current study, the results confirmed that an increase in detection spacing (i.e., 5-5-5; L_DD_ = 5 mm) will improve the measurement accuracy with error in an acceptable range (i.e., within 10%) over the peripheral vessel diameters (i.e., D = 4–7 mm) and physiologically relevant surrounding tissue conditions (transverse electrical conductivity of skeletal muscle tissue = 0.04–0.15 S/m, [6,10–16]). It should be noted that the simulations/experiments parameters were chosen to optimize guidewire specifications under conservative supra-physiological electrical conductivity conditions (i.e., 0.1%– 0.2% saline solution corresponding to 0.178–0.356 S/m).

As shown in previous studies [2–6,17,18], the two injection approach is a simple and effective methodology that allows easy and accurate lumen sizing of blood vessels based on the physics-derived analytical equations. The underlying assumption of the approach is that parallel conductance with different saline injections (e.g., normal and half normal saline solutions) is invariant and cancels out so that the CSA is determined only by the changes in total conductance and conductivity (Eq 2A). This assumption may not hold, however, if the parallel conductance becomes excessive (i.e., > 90%, Fig 2A).

As the outer-excitation electrodes are further apart to accommodate sizing of larger vessels, the determination of lumen diameter of smaller vessels may become less accurate because of the increased parallel conductance. The current results demonstrate this issue when the parallel conductance becomes very high relative to lumen conductance (i.e., a smaller lumen diameter plus higher electrical conductivity of surrounding tissue) while this becomes insignificant as lumen conductance predominates in larger lumen diameters (Figs 2 and 5). This is especially important for the peripheral arteries since the excitation spacing is configured to accommodate larger lumen diameter (i.e., D = 4–10 mm) based on the L_EE_ > 2D rule [3,7,19].

In the present study, an optimization of detection electrodes spacing was proposed to address the high parallel conductance due to the larger excitation spacing. Impact of the increased detection spacing on the diameter error was shown to be significant when the parallel conductance was too high (Figs 2A and3A). This impact was shown to be insignificant when the lumen conductance predominates over the parallel conductance (Fig 3B and 3C). There is a limit to the increase of the detection spacing, however, which can lead to over estimation of lumen diameter (Fig 3B).

The results show that a guidewire configuration with a certain range of detection-to-excitation ratios (i.e., L_DD_/L_EE_ = 0.2–0.3) can lead to a minimum error in the lumen diameter measurements (< 10%) for the majority of guidewire-lumen-tissue conditions considered (Figs 3 and 4). Our previous study^6^ showed that the measurement error has a strong dependency on the transverse electrical conductivity of skeletal muscle which surrounds the peripheral artery and monotonically decreases as the transverse electrical conductivity decreases. Since the range of transverse electrical conductivity of skeletal muscle reported corresponds to < 0.2 S/m (i.e., σ_T_ = 0.04–0.15 S/m, [6,10–16]), the present results suggest that the guidewire configuration of 5-5-5 (i.e., L_DD_ = 5 mm and L_DD_/L_EE_ = 0.3) can serve as an optimal design for determination of the lumen diameter over various anatomical configurations of the peripheral artery. This conclusion was also verified by *ex-vivo* measurements (Fig 5). Also, the conductance guidewire demonstrated better accuracy than US and QA in measuring phantom devices *in vivo* (Fig 6) which supports the performance and clinical relevance of the current electrode spacings. The conductance method showed no statistically significant bias and mean error < 4% which compares favorably to both US and QA determined in vivo sheath dimensions. US and QA (two routine imaging modalities clinically used to determine the vessel lumen diameter) showed a significant bias and errors as high as 10% and 17%, respectively. Hence, the conductance method was found to be more accurate than US and QA for the range of sheath dimensions considered.

For blood vessels, the Bland-Altman analysis for the pairwise comparison *in vivo* (Fig 7A and 7B) for conductance-US and QA-US showed that measurements are more scattered and biased for QA-US than conductance-US. Given the higher accuracy of conductance method over US and QA for the range of sheath dimension, theses *in-vivo* measurements support the performance superiority of the conductance method.

In summary, a design of the electrodes spacing is required for a given conductance guidewire or guidewire given its intended application (coronary, peripheral, etc.). Since the spectrum of anatomical and surrounding tissue features of the peripheral artery in patients are complex and diverse, it is imperative that the present findings are verified under clinical settings. Given that diseased peripheral vessels tend to be superficial and have atherosclerosis, the parallel conductance is expected to be small and the current physics-based guidance is expected to perform well clinically for the vessels of interest (proximal to popliteal arteries). A future development of a smaller guidewire (.018” or 0.014”) with more closely spaced electrodes can be used for tibials and other smaller arteries.

## Limitations of Study

The surrounding tissue of peripheral vessels is characterized as heterogeneous anatomical structures and anisotropic electrical properties. In the present study, the device-vessel-tissue configuration in computer simulations and *ex-vivo* measurements was simplified such that the heterogeneous structure and electrophysiological behavior of the tissue was considered as a lumped tissue with an isotropic electrical conductivity. Indeed, it has already been shown in our previous study [6] that despite the variability of conductance measurement accuracy by a wide variety of anatomical structures and electrical properties of the tissue, relatively large lumen diameter can play a favorable role in measurement accuracy for peripheral arteries due to the predominance of luminal conductance over parallel conductance. Furthermore, *in-silico* and *ex-vivo* observations from the current approach was shown to be consistent with and supported by *in-vivo* measurements performed where physiologically-relevant structure and function of surrounding tissue including anisotropic electrical properties are taken into account. Therefore, although distributive models of the surrounding tissue might lead to potential modifications of measurement accuracy, the increased detection spacing approach in the present study may play an important role in extending applicability of the conductance catheter for peripheral vessel diameter measurement.
